# Polyethylene glycol-coated haemostatic patch for prevention of clinically relevant postoperative pancreatic fistula after pancreatoduodenectomy: randomized clinical trial

**DOI:** 10.1093/bjsopen/zrad028

**Published:** 2023-04-06

**Authors:** Mario Serradilla-Martín, Sandra Paterna-López, Ana Palomares-Cano, Miguel Cantalejo-Díaz, Teresa Abadía-Forcén, Marta L Gutiérrez-Díez, Consuelo Artigas-Marco, Alejandro Serrablo-Requejo

**Affiliations:** Department of Surgery, Instituto de Investigación Sanitaria Aragón, Miguel Servet University Hospital, Zaragoza, Spain; Department of Surgery, Miguel Servet University Hospital, Zaragoza, Spain; Department of Surgery, Miguel Servet University Hospital, Zaragoza, Spain; Department of Surgery, Miguel Servet University Hospital, Zaragoza, Spain; Department of Surgery, Miguel Servet University Hospital, Zaragoza, Spain; Department of Surgery, Miguel Servet University Hospital, Zaragoza, Spain; Department of Surgery, Miguel Servet University Hospital, Zaragoza, Spain; Department of Surgery, Miguel Servet University Hospital, Zaragoza, Spain

## Abstract

**Background:**

The potential of haemostatic patches to reduce the rate of postoperative pancreatic fistula remains unclear. The aim of this trial was to evaluate the impact of a polyethylene glycol-coated haemostatic patch on the incidence of clinically relevant postoperative pancreatic fistula after pancreatoduodenectomy.

**Methods:**

In this randomized, single-centre, clinical trial, patients undergoing pancreatoduodenectomy were randomized 1 : 1 to receive pancreatojejunostomy reinforced with two polyethylene glycol-coated haemostatic patches (patch group) or without any reinforcement (control group). The primary outcome was clinically relevant postoperative pancreatic fistula, defined as grade B/C according to International Study Group of Pancreatic Surgery criteria, within 90 days. Key secondary outcomes were length of hospital stay, total rate of postoperative pancreatic fistula, and overall complication rate.

**Results:**

From 15 May 2018 to 22 June 2020, 72 patients were randomized, and 64 were included in the analyses (31 in the patch group and 33 in the control group). The risk of clinically relevant postoperative pancreatic fistula was reduced by 90 per cent (OR 0.10, 95 per cent c.i. 0.01 to 0.89, *P* = 0.039). Moreover, the use of the polyethylene glycol-coated patch retained its protective effect on clinically relevant postoperative pancreatic fistula in a multivariable regression model, significantly reducing the risk of clinically relevant postoperative pancreatic fistula by 93 per cent (OR 0.07, 95 per cent c.i. 0.01 to 0.67, *P* = 0.021), regardless of patient age, sex, or fistula risk score. The incidence of secondary outcomes did not significantly differ between the groups. One patient died within 90 days in the patch group *versus* three patients in the control group.

**Conclusions:**

A polyethylene glycol-coated haemostatic patch reduced the incidence of clinically relevant postoperative pancreatic fistula after pancreatoduodenectomy.

**Registration number:**

NCT03419676 (http://www.clinicaltrials.gov).

## Teaser

Hemopatch™ significantly reduced the incidence of clinically relevant postoperative pancreatic fistula in patients who underwent a pancreatoduodenectomy. The effectiveness of the patch was related to the fistula risk score.

## Introduction

Postoperative pancreatic fistula (POPF) is a common condition with incidences ranging from 13 to 41 per cent, and remains the most important and challenging determinant of postoperative morbidity and mortality after pancreatoduodenectomy (PD)^1–4^. POPF is critically associated with different surgical life-threatening complications, including sepsis, intra-abdominal abscesses, and postoperative haemorrhage, which prolong hospital stay and increase healthcare costs^3–5^.

Identifying patients at risk of having POPF, as well as predicting its development, may be crucial for preventing the incidence of severe sequelae. Different factors have been considered to influence the development of POPF, including soft pancreas, small main pancreatic duct diameter, high-risk pathology, and excessive blood loss^5,6^. The fistula risk score (FRS) was developed in an attempt to predict the risk of clinically relevant postoperative pancreatic fistula (CR-POPF)^5^.

Despite improvements in perioperative management and surgical techniques, aiming to decrease the incidence of POPF formation through the use of somatostatin analogues, pancreatic stents, minimally invasive surgery, reinforcement of the anastomosis with glue, autologous tissues, non-autologous absorbable or non-absorbable materials, no differences in POPF rates have been reported over the last decade^3,4,7–10^. This issue has been attributable to the fact that the underlying mechanism of POPF has not been fully elucidated, and other factors different from mechanical alterations of the pancreatoenteric anastomosis are involved in the process^11^.

Among the different approaches for preventing the onset of POPF, the use of sealants has emerged as a promising strategy^12^. Although the use of fibrin sealants and acrylic glues represents an attractive strategy for decreasing the risk of POPF, their benefits are uncertain for preventing the onset of POPF in patients undergoing PD^13–18^. Among the different sealants, a polyethylene glycol (PEG)-coated patch has been associated with positive outcomes in different surgical procedures^19–23^.

The purpose of this study was to evaluate the impact of a PEG-coated haemostatic patch on the incidence rate of CR-POPF after PD according to the International Study Group of Pancreatic Surgery (ISGPS) criteria^24^.

## Methods

### Study design

This study was conducted in accordance with the Declaration of Helsinki (as revised in 2013), performed according to CONSORT guidelines^25^, and Good Clinical Practice was followed. It was designed as a single-centre, open-label, parallel, two-arm, superiority, RCT in a tertiary-care centre in Zaragoza, Spain (Miguel Servet University Hospital), specializing in hepato-pancreato-biliary surgery, which had an annual frequency of more than 50 PD. The first patient was included on 15 May 2018 and the last one on 22 June 2020. A follow-up interval of 90 days was established to collect morbidity and mortality data. Follow-up visits were scheduled at 1 and 3 months after surgery, including CT imaging. No patient was withdrawn early and no patient was lost to follow-up. The study protocol was approved on 13 September 2017 by the Research Ethics Committee of the Community of Aragon, Spain (C.I. EC17/0062), and was registered (http://www.clinicaltrials.gov; NCT03419676). No changes to methods were done after trial commencement. The full trial protocol can be found in the *Supplementary material*.

### Participants

This study assessed for eligibility consecutive patients selected for undergoing PD with benign or malignant periampullary tumours or benign diseases (that is chronic pancreatitis) with a pancreatojejunostomy (PJ) reconstruction, male and female patients, between 18 and 80 years of age, with an ASA Physical Status Classification System^26^ grade of I–III. In the case of patients with pancreatic adenocarcinoma, only patients with initially resectable tumours were included. All patients underwent PD using an open approach.

Patients were excluded if they had any contraindication for performing PD, had acute necrotizing pancreatitis, had immune suppression, were unwilling to comply with the investigators and protocol indications, were incapable of providing written consent, had an ASA grade of greater than or equal to IV, had received neoadjuvant treatment, and required vascular resection during surgery. Data were collected and managed using Research Electronic Data Capture (REDCap, Vanderbilt University, Nashville, TN, USA). The patient cases were discussed in a multidisciplinary committee and surgery procedure was decided. Subsequently, once the patient was informed by any of the three surgeons responsible for performing the surgeries and had signed the informed consent, they were recruited for the trial.

### Surgical procedure

A standard open PD, using a reconstruction in two loops and a PJ, was performed by three surgeons with more than 10 years of experience in pancreatic surgery. Operative technique, patch placement, and drainage were standardized between the three surgeons. PD was performed without pylorus preservation. The pancreatic head and uncinate process were resected beyond the portal vein, and the resection was extended into the retroperitoneal, peripancreatic, pericaval, and interaortocaval lymphatic–fatty tissue with regional lymphadenectomy. For reconstruction, one jejunal limb was moved upwards behind the transverse colon and a PJ was constructed as the first anastomosis, followed by a Roux-en-Y hepatojejunostomy anastomosis. Another jejunal limb was used to perform the gastrojejunostomy in an antecolic position. Finally, a jejunojejunal anastomosis was performed. The PJ was performed in one layer using a continuous barbed suture (V-Loc™ 3/0; Medtronic, Dublin, Ireland) in the posterior and anterior sides of the anastomosis or using a two-layer, duct-to-mucosa, end-to-side technique, with interrupted sutures using monofilament sutures, according to the individual surgeon’s preference. A 5- or 8-Fr internal stent was always placed in the PJ, depending on the diameter of the main pancreatic duct.

Different risk factors associated with the onset of POPF^5^ (small duct, soft pancreas, high-risk pathology, and excessive blood loss) were evaluated during PD and were used to calculate a 10-point FRS. According to this FRS classification system, the patients were stratified as follows: negligible risk, 0 points; low risk, 1–2 points; intermediate risk, 3–6 points; and high risk, 7–10 points^5^.

### Intraoperative and postoperative management

According to the Enhanced Recovery After Surgery guideline recommendations for PD^27^, thoracic epidural anaesthesia was performed to improve analgesia and a nasogastric tube was not used in these patients per protocol. In addition to postoperative nausea and vomiting prophylaxis, venous thromboembolism prophylaxis 6 h after surgery was commenced. The patients were mobilized the same afternoon of the intervention and a progressive diet was started on postoperative day 1. In those patients in whom the amylase concentration in the drains was less than three times the institutional upper normal serum level at 24 h after the intervention, the drain was removed before 72 h. The urinary catheter was removed on postoperative day 1.

### Intervention

In the patch group, two PEG-coated, collagen-based, haemostatic patches (Hemopatch™ Sealing Hemostat, Baxter Aktiengesellschaft, Vienna, Austria) with a size of 9 × 4.5 cm per patch were used in each patient. The objective was to place one patch underside and another one anterior, once the anastomosis was completed, according to the manufacturer’s instructions. Patches were usually placed in the absence of bleeding, although traces of blood from the surgical field may be enough to initiate activation of the patch PEG coating. In the case of an insufficient amount of blood, a few drops of sodium bicarbonate were applied over the dry tissue surface before application of the patch, increasing the covalent bonds between the tissue and the active surface of the patch and thus improving adherence. The PJ was left bare in the control group.

### Use of drains and postoperative pancreatic fistula assessment

In both groups, a silastic Penrose^®^ drain (Cardinal Health, Dublin, OH, USA) was placed on the posterior side of the PJ from the left side of the abdominal wall. A Blake^®^ drain (Ethicon, Raritan, NJ, USA) was placed on the right side under the hepatojejunostomy. To evaluate the presence of POPF, amylase, and/or lipase enzymes, determinations were performed in the serum and perianastomotic drainage fluid at days 3, 5, and 7, and then every 2 days (if drainage was maintained). In the absence of drainage, a diagnostic imaging tool, either ultrasound or CT, was used to confirm or exclude the presence of POPF. Each drain was removed when the amylase concentration was less than three times the institutional upper normal serum level (100 U/l). In asymptomatic patients with persisting high levels of amylase, the drains were removed when the volume was less than 20 ml per day.

### Outcome measures

The primary outcome was the incidence rate of CR-POPF within 90 days defined according to the ISGPS criteria^24^. Secondary outcomes were length of hospital stay, length of ICU stay, total rate of POPF (including biochemical fistula), reoperation rate (including interventional radiology), delayed gastric emptying^28^, bile leak^29^, postoperative haemorrhage^30^, deep or organ/space infection^31^, death (regardless of the cause), and overall complication rate up to 90 postoperative days (according to Clavien–Dindo classification^32^).

### Sample size

An observational study was carried out for patients undergoing PD in our institution and indicated a baseline 31 per cent CR-POPF rate. According to power calculations, 62 patients (31 per group) were required to show a decrease in CR-POPF rate from 31 to 5 per cent with 80 per cent power and two-tailed 5 per cent significance level, with assumed 10 per cent lost to follow-up.

### Randomization

Patients were randomized on a 1 : 1 basis during surgery by the minimization method (preferred treatment probability 0.9), after resection but before anastomosis, to either PEG-coated patch application (patch group) or no patch (control group). Randomization was done by blocks of 10 patients, using a secure, internet-based, randomization service (Randomizer^®^), provided by the Centre of Medical Statistics, Medical University of Graz, Austria^33^, to all participating sites. Randomization was stratified by pancreatic gland texture (soft or normal), pancreatic duct diameter (up to 3 mm or larger than 3 mm), and intraoperative bleeding (up to 700 ml or more than 700 ml).

Patients were assigned to one of the following study groups: patch group, patients undergoing PD with PJ reinforced with two PEG-coated collagen-based haemostatic patches; and control group, patients undergoing PD with PJ without any sealant reinforcement. Although operating-room staff and doctors responsible for the follow-up were not blind to the intervention, data analysis was performed in a masked fashion.

### Statistical analysis

Statistical analyses were performed using R software (version 4.0.3)^34^. For descriptive statistics, the number (percentage) or median (i.q.r.) was used for categorical and continuous variables respectively. Data were tested for normal distribution using the Shapiro–Wilk test. The χ^2^ test with Yates correction or the independent-sample Mann–Whitney *U* test was used for comparing categorical and continuous variables respectively.

A multivariable logistic regression model was used to estimate the OR and the 95 per cent c.i. associated with the use of patches and CR-POPF. POPF survival rates were plotted for treatment groups using Kaplan–Meier analysis and were compared using a log rank test. A *P* value of <0.050 was considered significant, and all tests were two-tailed.

## Results

**Fig. 1:**
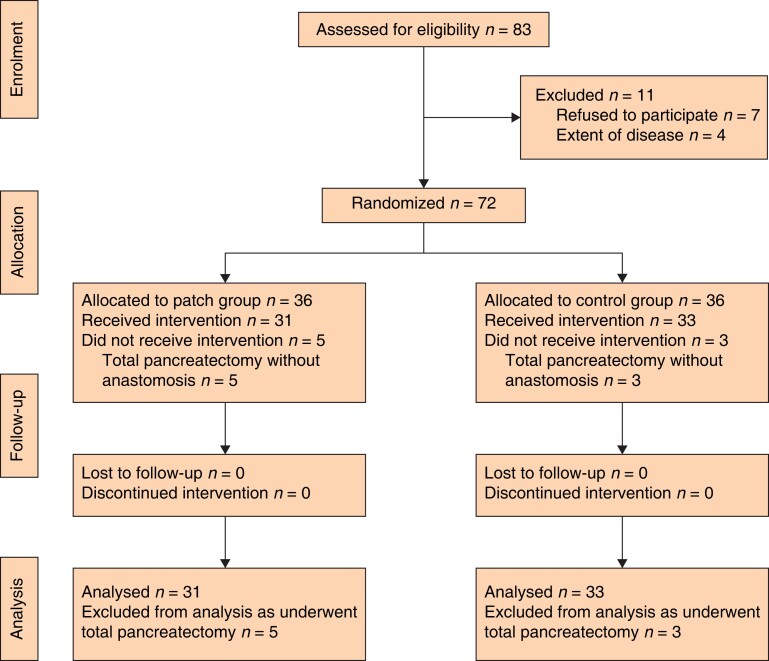
CONSORT diagram

Among the total 72 patients who were randomized (36 in each group), five subjects in the patch group and three in the control group underwent a total pancreatectomy and were excluded from the analyses. The final analyses included 64 patients (31 in the patch group and 33 in the control group) (*Fig. 1*). At baseline, no significant differences were observed in demographic and clinical characteristics between the patch and control groups (*Table 1*).

**Table 1:** Baseline clinical and demographic characteristics

	Overall (*n* = 64)	Patch group (*n* = 31)	Control group (*n* = 33)
**Male : female**	41 (64) : 23 (36)	19 (61) : 12 (39)	22 (67) : 11 (33)
**Age (years), median (i.q.r.)**	69.0 (59.9–74.5)	69.9 (60.5–76.3)	67.7 (60.1–73.6)
**BMI (kg/m^2^), median (i.q.r.)**	25.0 (22.6–29.0)	25.0 (22.5–28.1)	25.4 (23.0–30.1)
**Diabetes**			
Yes	19 (30)	8 (26)	11 (33)
No	45 (70)	23 (74)	22 (67)
**Preoperative biopsy**			
Yes	40 (63)	20 (65)	20 (61)
No	24 (37)	11 (35)	13 (39)
**Preoperative biliary stenting**	45 (70)	21 (68)	24 (73)
**Diagnosis**			
PDAC	37 (58)	19 (61)	18 (55)
Metastasis	1 (2)	1 (3)	0 (0.0)
MCN	5 (8)	3 (9)	2 (6)
IPMN	3 (5)	1 (3)	2 (6)
Chronic pancreatitis	3 (5)	2 (7)	1 (3)
NET	6 (9)	4 (12)	2 (7)
Other	9 (14)	3 (9)	6 (18)

Values are *n* (%) unless otherwise indicated. PDAC, pancreatic ductal adenocarcinoma; MCN, mucinous cystic neoplasm; IPMN, intraductal papillary mucinous neoplasm; NET, neuroendocrine tumours.

Surgical time was shorter in the patch group (median 265 *versus* 305 min, *P* = 0.002), whereas other intraoperative variables did not show any difference between the patch and control groups (*Table 2*). When analysing the distribution of surgical times and the use of the PEG-coated haemostatic patch, the authors appreciate that the difference was only observed in patients with an intermediate–high risk of POPF according to the FRS. In these patients, there is less blood loss in the group with the PEG-coated haemostatic patch (*P* = 0.007), which presupposes fewer complex surgeries and, therefore, less operative time.

**Table 2:** Overview of the intraoperative characteristics of the study sample

	Overall (*n* = 64)	Patch group (*n* = 31)	Control group (*n* = 33)
**Surgical time (min), median (i.q.r.)**	300 (258–355)	265 (232–332)	305 (300–380)
**DTMA**			
Interrupted	8 (13)	3 (10)	5 (15)
Continuous	56 (87)	28 (90)	28 (85)
**Pancreas texture**			
Hard	25 (39)	14 (45)	11 (33)
Soft	39 (61)	17 (55)	22 (67)
**PDD (mm), median (i.q.r.)**	3.0 (2.0–5.0)	3.0 (2.0–4.0)	3.0 (2.0–5.0)
**Intraoperative bleeding (ml), median (i.q.r.)**	200 (158–300)	200 (115–225)	200 (170–300)
**FRS**			
Median (i.q.r.)	7.38 (5.30–7.76)	7.32 (5.79–7.61)	7.41 (5.40–7.94)
Negligible	0 (0)	0 (0)	0 (0)
Low	18 (28)	8 (26)	10 (30)
Intermediate	42 (66)	23 (74)	19 (58)
High	4 (6)	0 (0)	4 (12)

Values are *n* (%) unless otherwise indicated. DTMA, duct-to-mucosa anastomosis; PDD, pancreatic duct diameter; FRS, fistula risk score.

POPF rate, including biochemical leakage, was 38 per cent (24/64 patients) in the overall study sample, with nine (14 per cent) patients presenting grade B/C POPF. Regarding the overall POPF rate, there was no significant difference between the patch group (29 per cent) and the control group (52 per cent) (*Table 3*). The CR-POPF rate was significantly lower in the patch group (3 per cent) than in the control group (24 per cent) (*Table 3*). Similarly, the authors analysed the pancreatic fistula rate according to the technique used (continuous suture *versus* interrupted suture), with no statistically significant differences in the overall rate (*P* = 0.699) or in the CR-POPF rate (*P* = 0.587) (*Table S1*).

**Table 3:** Overview of the secondary outcomes of the study within 90 days after surgery in the overall study population, the patch group, and the control group

	Overall (*n* = 64)	Patch group (*n* = 31)	Control group (*n* = 33)	*P*	OR (95% c.i.)
**Hospital stay (days), median (i.q.r.)**	14.5 (11.0–25.2)	14.0 (11.0–23.5)	15.0 (12.0–28.0)	0.408*	0.99 (0.96 to 1.02)
**ICU stay (days), median (i.q.r.)**	3.0 (2.0–5.0)	3.0 (2.0–3.5)	4.0 (2.0–5.0)	0.110*	0.95 (0.88 to 1.03)
**POPF**					
Total	24 (38)	7 (29)	17 (52)	0.033†	0.28 (0.09 to 0.82)
Biochemical leakage	15 (24)	6 (26)	9 (27)	0.893†	0.65 (0.19 to 2.11)
CR-POPF	9 (14)	1 (3)	8 (24)	0.027†	0.12 (0.00 to 0.73)
**Reoperation rate‡**	14 (22)	6 (19)	8 (24)	0.639*	0.52 (0.14 to 1.77)
Interventional radiology	6 (9)	3 (10)	3 (9)	0.928*	1.00 (0.10 to 9.61)
Surgery	10 (16)	4 (13)	6 (18)	0.618*	0.44 (0.07 to 2.66)
**DGE**	10 (16)	4 (13)	6 (18)	0.734*	0.68 (0.15 to 2.73)
**Bile leak**	10 (16)	5 (16)	5 (15)	1.000*	1.08 (0.26 to 4.44)
**Haemorrhage**	11 (17)	6 (19)	5 (15)	0.909*	1.33 (0.35 to 5.31)
**DOS**	10 (16)	3 (10)	7 (21)	0.305*	0.41 (0.08 to 1.71)
**Mortality**	4 (6)	1 (3)	3 (9)	0.614*	0.37 (0.01 to 3.36)
**Overall complication rate**	63 (98)	30 (97)	33 (100)	0.484*	§
**Major complications¶**	11 (17)	4 (13)	7 (21)	0.583*	0.56 (0.13 to 2.15)
**Readmission**	14 (22)	5 (16)	9 (27)	0.438*	0.52 (0.14 to 1.77)

Values are *n* (%) unless otherwise indicated. *Mann–Whitney *U* test. †χ^2^ test. ‡Number of patients reoperated on, including interventional radiology, surgery, or both. §OR and 95% c.i. cannot be estimated. ¶Defined as Clavien–Dindo greater than or equal to IIIa^32^. OR, odds ratio; POPF, postoperative pancreatic fistula; CR-POPF, clinically relevant postoperative pancreatic fistula; DGE, delayed gastric emptying; DOS, deep or organ/space infection.

The risk of CR-POPF was reduced by 90 per cent (OR 0.10, 95 per cent c.i. 0.01 to 0.89, *P* = 0.039) (unadjusted model; *Table 4*). Moreover, the use of the PEG-coated patch retained its protective effect on CR-POPF in a multivariable regression model, integrating the most important risk factors for CR-POPF (age, sex, and FRS). According to the latter model, the use of the PEG-coated patch significantly reduced the risk of CR-POPF by 93 per cent (OR 0.07, 95 per cent c.i. 0.01 to 0.67, *P* = 0.021) regardless of patient age, sex, or FRS (model 2; *Table 4*).

**Table 4:** Unadjusted and adjusted OR of the role of the polyethylene glycol-coated haemostatic patch in the risk of clinically relevant-postoperative pancreatic fistula

	Unadjusted	Model 1	Model 2
**PEG-coated patch**			
OR	0.10	0.06	0.07
95% c.i.	0.01,0.89	0.01,0.64	0.01,0.67
*P*	0.039	0.020	0.021

Model 1: adjusted by age and sex. Model 2: adjusted by age, sex, and fistula risk score (low *versus* intermediate/high). OR, odds ratio; PEG, polyethylene glycol.

The length of hospital stay and length of ICU stay were similar in the patch and control groups. The incidence of other secondary outcomes did not significantly differ between the study groups (*Table 3*), although there was a downward trend in the rate of delayed gastric emptying, deep or organ/space infection, and readmission rate, favouring the patch group compared with the control group. The 90-day mortality rate was 3 per cent in the patch group and 9 per cent in the control group (*Table 3*). There were no missing values in either primary or secondary outcome measurements.

## Discussion

The results of this study suggested that in patients who underwent PD with a PJ, the use of the PEG-coated collagen patch reduces the incidence of CR-POPF. Moreover, the capacity of the PEG-coated patch to prevent the onset of POPF was dependent on the FRS: the higher the FRS, the higher the protection. The incidences of adverse events were lower in the patch group compared with the control group, although no significant differences were observed in the specific postoperative complications analysed.

Although different surgical approaches are currently used for PD, there does not seem to be an optimal technique that significantly decreases POPF rates^11^. Despite postoperative mortality rates having significantly decreased to less than 5 per cent, PD may still be associated with relevant morbidity, mainly related to POPF^1–4,9,35,36^.

From a clinical point of view, POPF may be divided into ‘clinically relevant’ (grade B/C) and ‘not clinically relevant’ (biochemical leakage)^24,37^. CR-POPF represents postoperative complications that may significantly impact clinical outcomes. Whereas grade B POPF may be conservatively managed, grade C POPF requires in most patients a surgical review, generally associated with a high level of morbidity and mortality^24,38^.

The current study reported an overall POPF rate of 38 per cent (24/64 patients), which lies within the range of the published series^1–4,38^. Additionally, the rate of CR-POPF was significantly lower in the patch group than in the control group.

As far as the authors know, this is the first RCT evaluating the effectiveness of a PEG-coated haemostatic patch in PD, which makes it difficult to compare these results with the current evidence. Pisapia *et al*.^39^, in a retrospective study, found lower incidence rates of POPF in patients who underwent distal pancreatectomy with a PEG-coated haemostatic patch. Similarly, Uranues *et al*.^40^ reported that the use of a PEG-coated haemostatic patch was associated with significantly lower rates of CR-POPF in patients with hand-sewn stump closure and in those where the main pancreatic duct was ligated selectively.

The decrease in CR-POPF rate observed in this study was not associated with a significant reduction in the incidence of other specific postoperative outcomes or a lower mortality rate.

The use of sealants may be an option to reduce the incidence of POPF and, therefore, reducing life-threatening complications and healthcare costs^3,4^. Although the use of different sealants and acrylic glues has not provided unequivocal data on their ability to reduce the incidence of POPF in patients undergoing PD^3,13–17^, relevant differences in the study protocol, sealant characteristics, and surgical procedures/techniques may have a significant impact on the outcomes. Moreover, current scientific evidence suggests that, in patients who undergo a PD, fibrin sealants make little to no difference to the POPF rate or mortality compared with control^4^.

No evidence is currently available regarding the association of the use of the fibrin sealant patch (TachoSil™) in patients who undergo a PD with a lowering of the incidence of POPF or a reduction of its severity^3,18^. Schindl *et al*.^3^ assessed in a multicentre trial whether the use of a fibrin sealant patch was associated with lower incidence rates of POPF in patients who underwent PD with PJ. According to the results of this study, the incidence of CR-POPF did not significantly differ between patients who underwent surgery with the fibrin sealant patch and those who did without^3^. Similarly, the ability of a fibrin sealant patch to reduce the incidence of POPF after pancreatoenteric anastomosis was evaluated in a prospective and randomized study. Of the total 124 patients included in the study, CR-POPF was observed in four patients (7 per cent) in both the intervention and control groups, with no significant differences (*P* = 1.000) observed in any of the secondary outcomes^18^.

Hemopatch™ is a PEG-covered collagen matrix, which has been proven to be an effective haemostat and sealant in different surgical procedures^19–23,39,40^. The rationale for its use is based on the hypothesis that it may reduce the overflow of pancreatic juice from the anastomosis during the early postoperative interval and, unlike other fibrin sealants, it may resist the degrading effect of the enzymes due to its structure, a collagen pad coated with a thin layer of a four-armed cross-linking agent (a rapid protein-reactive monomer^41^), which provides advantages compared with human-derived proteins^42,43^.

This study has some limitations that need to be taken into consideration. The main one is that it is a single-centre study, which included a small number of patients. The lack of differences in secondary outcomes may be due to the fact that the study was underpowered for detecting such differences. This study only used one type of reconstruction technique and appropriate caution is therefore recommended when extending the results to other reconstruction techniques. Finally, it should be mentioned that the use of interrupted suture in eight patients may have biased the results. Nevertheless, the incidence rate of POPF was similar for both suture techniques.

Further research is needed to elucidate the role of different baseline factors in the effectiveness of the patch and the incidence of CR-POPF in larger, prospective multicentre controlled trials.

## Supplementary Material

zrad028_Supplementary_Data

## Data Availability

The datasets generated during and/or analysed during the current study are available from the corresponding author on reasonable request.
