# Supplementary material for: Polyethylene glycol-coated haemostatic patch for prevention of clinically relevant postoperative pancreatic fistula after pancreatoduodenectomy: randomized clinical trial
Source: BJS Open. 2023 Apr 6;7(2):zrad028. doi: 10.1093/bjsopen/zrad028 (PMC10077024; doi:10.1093/bjsopen/zrad028)
Supplement: zrad028_Supplementary_Data [file zrad028_supplementary_data.docx]

**Title**

**PEG-coated haemostatic patch for prevention of clinically relevant postoperative pancreatic fistula after pancreatoduodenectomy: randomized clinical trial**

**Authors**

Mario Serradilla-Martín, MD, PhD^1^, Sandra Paterna-López, MD, PhD^2^, Ana Palomares-Cano, MD^2^, Miguel Cantalejo-Díaz, MD^2^, Teresa Abadía-Forcén, MD^2^, Marta L. Gutiérrez-Díez, MD, PhD^2^, Consuelo Artigas-Marco, MD, PhD^2^, Alejandro Serrablo-Requejo, MD, PhD^2^

^1^Instituto de Investigación Sanitaria Aragón, Department of Surgery, Miguel Servet University Hospital, Zaragoza, Spain

^2^Department of Surgery, Miguel Servet University Hospital, Zaragoza, Spain

**Corresponding author:**

Mario Serradilla-Martín
Department of Surgery
Miguel Servet University Hospital
Paseo Isabel La Católica, 1-3
50009 Zaragoza (Spain)

E-mail: [marioserradilla@hotmail.com](about:blank)

ORCID: 0000-0002-2466-0711

Twitter: @MarioSerradilla2

**Supplementary Materials - Index**

| **Supplementary Methods** |  |
| --- | --- |
| Study Protocol | *Page 3* |
| **Supplementary Tables** |  |
| Table S1 | *Page 21* |
|  |  |

**Supplementary Methods**

**STUDY PROTOCOL.**

# STUDY PROTOCOL AND STATISTICAL ANALYSIS PLAN

**Use of Hemopatch as a sealant in the pancreatojejunostomy after pancreatoduodenectomy to prevent postoperative pancreatic fistula**

# Mario Serradilla Martín

**Division of Hepato-Pancreato-Biliary Surgery Department of Surgery**

# Miguel Servet University Hospital Zaragoza, Spain

Version 3.0 25/01/2018

This document is confidential and can not be circulated without the written authorization of the principal investigators.

**Content**

1. [Synopsis of the study. 3](#_TOC_250021)
2. Visit and evaluation. 5
3. [Structure of the organization 5](#_TOC_250020)
   1. [Sponsor… 5](#_TOC_250019)
   2. [Principal investigators… 5](#_TOC_250018)
4. [Summary… 7](#_TOC_250017)
5. Hypotheses / objectives 9
6. [Study design… 9](#_TOC_250016)
   1. [Type of study 9](#_TOC_250015)
   2. [Primary objective and secondary objectives 9](#_TOC_250014)
   3. Duration of study 10
7. [Patients. 10](#_TOC_250013)
   1. [Criteria. 10](#_TOC_250012)
   2. [Recruitment… 11](#_TOC_250011)
   3. [Inclusion and exclusion criteria 11](#_TOC_250010)
   4. Surgical techniques and materials of intervention 11
   5. [Postoperative period 12](#_TOC_250009)
   6. [Complications / failure of the intervention 13](#_TOC_250008)
   7. Termination conditions / Adverse events 13
8. [Data. 14](#_TOC_250007)
   1. Calculation of sample size 14
   2. [Randomization and groups. 14](#_TOC_250006)
   3. Evaluation of efficacy… 14
   4. [Patient data sheet… 15](#_TOC_250005)
   5. [Statistical methods and data analysis 15](#_TOC_250004)
9. [Patient safety… 16](#_TOC_250003)
10. Impact on practice 16
11. [Ethics and legal requirements 16](#_TOC_250002)
12. Registry… 16
13. [Publication. 16](#_TOC_250001)
14. [References. 17](#_TOC_250000)

# SYNOPSIS OF THE STUDY.

| **Principal investigator** | Mario Serradilla Martín, MD, FACS Associate Professor at University of Zaragoza Attending Surgeon in General Surgery Division of Hepato-Pancreato-Biliary Surgery Miguel Servet University Hospital  Paseo Isabel La Católica, 1-3 50009 Zaragoza, Spain  Tel.: +34 636 006 184  Fax: +34 976 765 509  E-mail: [marioserradilla@hotmail.com](mailto:marioserradilla@hotmail.com) / [mserradilla@salud.aragon.es](mailto:mserradilla@salud.aragon.es) |
| --- | --- |
| **Title of the study** | Use of Hemopatch as a sealant in the pancreaticojejunostomy after pancreatoduodenectomy to prevent postoperative pancreatic fistula |
| **Population** | Patients undergoing pancreatoduodenectomy (PD) (due to  benign or malignant tumors or chronic pancreatitis) with duct-to-mucosa pancreaticojejunostomy reconstruction |
| **Objetives** | **Primary objective:**   - To demonstrate the effectiveness of Hemopatch in the prevention of postoperative pancreatic fistula after PD with duct-to-mucosa reconstruction against duct-to-mucosa reconstruction without Hemopatch   **Secondary objectives:**   - To assess the safety and tolerance of Hemopatch in patients undergoing PD |
| **Interventions** | Performing duct-to-mucosa anastomosis with or without Hemopatch reinforcement after PD |
| **Inclusion and exclusion criteria** | **Inclusion criteria:** patients submitted to PD by open approach for benign/malignant periampullary tumours or benign; ASA < 4; men and women ≥ 18 and ≤ 80 years of age.  **Exclusion criteria:** patients under 18 and over 80 years old; ASA score ≥ 4; subjected to multivisceral resections different from PD; patients with acute necrotizing pancreatitis; immunosuppressed patients; patients who have not signed the informed consent; patient with  contraindication of PD performance; vascular reconstruction; neoadjuvant therapy. |
| **Results** | **Primary point:**  Type B and C postoperative pancreatic fistula defined according to the criteria of the International Study Group on Pancreatic Fistula (ISGPF)  **Secondary endpoints:**   1. Duration of postoperative hospital stay. 2. Length of stay in intensive care unit. 3. Reinterventions including interventional radiology. 4. Deep or organ space infections according to CDC criteria |

|  | 1. Delayed gastric emptying. 2. Biliary fistula. 3. Postoperative hemorrhage. 4. Death, regardless of the cause. |
| --- | --- |
| **Type of study** | Prospective controlled unicentric clinical trial |
| **Statistic analysis** | Statistical analyses will be performed using R software (version 4.0.3). Descriptive statistics number (percentage) or median [interquartile range] will be used for categorical and continuous variables, respectively. Data were tested for normal distribution using the Shapiro-Wilk test. The Chi^2^ with Yates correction or the independent sample Mann-Whitney U-test were used for comparing categorical and continuous variables, respectively.  A multivariable logistic regression model will be used to estimate the odd ratios and the 95% confidence intervals (CIs) associated with the use of patches and CR-POPF. Postoperative pancreatic fistula survival rates will be plotted for treatment groups using Kaplan–Meier analysis and were compared using a log-rank test. A p-value of less than 0.05 was considered significant, and all tests were two-tailed. |
| **Sample size** | An observational study was carried out for patients undergoing PD in our institution and indicated a baseline 31% CR-POPF rate. According to power calculations, 62 patients (31 per group) were required to show decrease of CR-POPF rate from 31% to 5% with 80% power and two tailed 5% significance level, with assumed 10% loss-to-follow-up. |
| **Ethical approval** | This study is approved by the Clinical Research Ethics Committee of Aragon (CEICA) |
| **Duration of the study** | The study will begin on February 1, 2018. It is estimated a  duration of 1.5 years, according to the PD rate of our center (40-50 year). Estimated end date: July 31, 2019. |
| **Participating centers** | Division of Hepato-Pancreato-Biliary Surgery, Department of Surgery, Miguel Servet University  Hospital |

# PATIENT VISIT AND EVALUATION.

There will be a first visit in an outpatient clinic where the patient will be informed about the study in detail and will be offered to participate in it as long as it meets the selection criteria. Two copies of the informed consent will be signed, one of them for the patient and the other will be kept together with the study documentation. A first evaluation of the patient will be carried out as it appears in the data collection notebook.

# STRUCTURE OF THE ORGANIZATION.

# Sponsor.

Investigator Initiated Research Grant from Baxter S.L., Chicago, Illinois, United States, convened on March 24, 2017.

# Principal investigators.

Principal investigator:

Mario Serradilla Martín, MD, FACS Associate Professor at University of Zaragoza Attending Surgeon in General Surgery Division of Hepato-Pancreato-Biliary Surgery Miguel Servet University Hospital

Paseo Isabel La Católica, 1-3 50009 Zaragoza, Spain

Tel .: +34 636 006 184

Fax: +34 976 765 509

E-mail: [marioserradilla@hotmail.com](mailto:marioserradilla@hotmail.com) / [mserradilla@salud.aragon.es](mailto:mserradilla@salud.aragon.es) Collaborating researchers:

Alejandro Serrablo Requejo, MD, PhD, FACS Associate Professor at University of Zaragoza

Head of Division, Division of Hepato-Pancreato-Biliary Surgery Department of Surgery

Miguel Servet University Hospital Paseo Isabel La Católica, 1-3 50009 Zaragoza, Spain

Tel .: +34 629 675 491

Fax: +34 976 765 509

E-mail: [almaley@telefonica.net](mailto:almaley@telefonica.net)

Sandra Paterna López, MD

Attending Surgeon in General Surgery Division of Hepato-Pancreato-Biliary Surgery Miguel Servet University Hospital

Paseo Isabel La Católica, 1-3 50009 Zaragoza, Spain

Tel .: +34 619 520 628

Fax: +34 976 765 509

E-mail: [pichicatos@yahoo.es](mailto:pichicatos@yahoo.es)

Methodological, Statistical and Documentation Consultant:

José Miguel Arbonés Mainar, PhD Aragonese Institute of Health Sciences Translational Research Unit

Miguel Servet University Hospital Paseo Isabel La Católica, 1-3 50009 Zaragoza, Spain

Tel .: +34 976 769 565

Fax: +34 976 769 566

E-mail: [jmarbones.iacs@aragon.es](mailto:jmarbones.iacs@aragon.es) Web: [www.adipofat.com](http://www.adipofat.com/)

# SUMMARY.

Pancreatoduodenectomy (PD) is a common surgical procedure to treat carcinoma of the head of the pancreas and other tumors of the periampullary region. Recent advances in surgical techniques and perioperative treatments have reduced the perioperative mortality below 10% in high volume centers [1–4]. However, PD is associated with considerable morbidity in 40–58.5% of patients manifesting postoperative pancreatic fistula (POPF), delayed gastric emptying (DGE), postpancreatectomy hemorrhage (PPH), and postoperative pulmonary complications [2, 5, 6].

Several surgical techniques and perioperative care have been studied in order to prevent or decrease the incidence of POPF after PD, including digestive tract reconstruction with pancreaticogastrostomy [7], duct-to-mucosa (DTM) or invagination pancreatojejunostomy (PJ) [8], prophylactic somatostatin or somatostatin analogues [9], and pancreatic duct stents [10].

Probably the reasons why the pancreatojejunal anastomosis has greater diffusion are different, starting from the first descriptions of the surgical technique with subsequent inertia, until it is feasible to use 3 anastomosis reconstruction after PD with the same jejunal handle. Currently, there is a growing pancreatic reconstruction trend by pancreaticogastrostomy technique. However, the results of prospective randomized studies as well as meta-analyzes conclude that both techniques are safe and that the rate of fistula is similar in both cases. Despite this, with the pancreaticogastrostomy technique, there is a higher rate of postoperative bleeding and a higher rate of DGE [11- 12]. There is another factor in the choice of the technique, which is the consistency of pancreatic remnant. A soft pancreas will always be an added risk for the onset of the fistula. In these cases, many surgeons choose a PJ according Peng’s technique [13].

Although perioperative morbidity and mortality associated with a PD has improved significantly over the years, even in high-volume centers, the incidence of POPF is still in the range of 9.9–28.5% [14–16]. Therefore, an ideal pancreatic reconstruction technique or perioperative treatment, which prevents POPF, is still unavailable.

The use of sealants has been one of the approaches performed in order to try to reduce the POPF (Table 1). Some non-controlled or non-randomized studies have shown that the use of fibrin glue based sealants in combinations with patches of felts may lead to a POPF grade B/C of 0-10% [18-21]. Only 2 randomized control trials have been performed with fibrin glue, with opposite results in terms of significant reduction of POPF [17, 22].

Table 1. Studies with the use of sealants in PD and rates of pancreatic fistula.

| **CONTRO**  **Author Year Study type Patients / N** | | | **L** | **TREATMENT** | | **P** |
| --- | --- | --- | --- | --- | --- | --- |
| **Surgery** | | Technique | POPF | Technique | POPF |  |
| High risk  Lillemoe 2004 RCT (soft  [17] panc/small  duct) | 125 | Ø (66) | 30%^1^ | Fibrin glue: Hemaseel (59) | 26%^1^ | NS |
| PGA felt + Ochiai 2010 Retrospective - 54 Ø (36) 38.9%^2^ Fibirnogen/  [18] thrombin  patch (18) | | | | | 5.6%^2^ | 0.016 |
| Pozzo Retrospective  [19] 2010 non- -  controlled | 27 | - | - | Tachosil | 0%^2^ | - |
| Retrospective Duct-to-  Mita [20] 2011 non- mucosa  controlled | 40 | - | - | Tachocomb | 7,5%^2^ | - |
| Prospective  Satoi [21] 2011 (historical -  control) | 128 | Ø (78) | 14%^2^ | PGA felt + Fibrin glue: Bolheal (50) | 10%^2^ | NS |
| Martin 2013 RCT - 57 Ø (32) 43.8%^1^ Fibrin glue:  [22] TISSEEL (59) | | | | | 40%^1^ | NS |
| 1- Fistula grade A, B & C; 2- Fistula grade B & C | | | | | | |

Hemopatch is an NHS-PEG patch that consists of a soft, thin, pliable, flexible pad of collagen derived from bovine dermis, coated with NHS-PEG (pentaerythritol polyethylene glycol ether tetra-succinimidyl glutarate). It is intended as a surgical sealant for procedures in which control of leakage by conventional surgical techniques is either ineffective or impractical [23], making it a plausible option to use during PD surgical technique in order to decrease POPF.

# Preliminary experience with Hemopatch: case serie

In our center we have been using this NHS-PEG patch envolving the pancreatojejunostomy as a new way to decrease POPF after PD. The results of more than a year of experience have been submitted as an abstract communication to the E- AHPBA congress in Mainz, Germany (May, 2017), and are the following:

# Sealing with NHS-PEG patch to prevent postoperative pancreatic fistula after pancreatojejunostomy

Mario Serradilla, Ana Palomares, Sandra Paterna, José Manuel Ramia, Alejandro Serrablo

Background. Postoperative pancreatic fistula (POPF) is a common and most severe complication following pancreatoduodenectomy (PD) (9.8% to 34.2%). POPF not only prolongs hospital stay and increases healthcare costs, but also plays a central role in the development of life-threatening events such as intra-abdominal abscess and

postoperative hemorrhage. We present a new way to decrease POPF after PD using a NHS-PEG patch envolving duct-to-mucosa (DTM) pancreatojejunostomy.

Materials and methods. 26 consecutive PD were performed from July 2015 to October 2016, using the same technique, 13 of them sealing with NHS-PEG patch after DTM. Both groups were statistically homogeneous. Demographic data were collected (age, gender, diagnosis, date of intervention), and rates of postoperative complications (pancreatic fistula, biliary fistula, delayed gastric emptying –DGE-, hemorrhage, exitus, Clavien-Dindo classification, readmission and mean stay).

Results. Postoperative complication rates were (with NHS-PEG patch/without NHS- PEG patch): pancreatic fistula (A: 7.7%/7.7%; B: 0%/7.7%; C: 0%/15.5%); biliary fistula: 7.7%/15.4%; DGE: 7.7%/7.7%; hemorrhage: 7.7%/15.5%; exitus: 0%/7.7%; readmission: 7.7%/46%; mean stay: 21.3/26.1 days; other complications: 61.5%/46.2%. Discussion. Sealing with NHS-PEG patch to prevent postoperative pancreatic fistula after DTM pancreatojejunostomy can offer a new possibility to decrease POPF, with less fistula rate B and C, less hospital stay an less healthcare costs. Randomized controlled trials with larger number of patients should be performed to support this theory.

Therefore, we have design the following randomized controlled trial in order to validate our positive experience with Hemopatch in PD.

# HYPOTHESIS / OBJECTIVE.

This unicentric, controlled, randomized study investigates the efficacy and tolerance of Hemopatch in the prevention of POPF type B and C after a PD. The goal for Hemopatch is to reduce the risk of POPF type B and C from 30% to 15%.

# STUDY DESIGN.

# Type of study.

Unicentric, randomized, controlled, open-label, prospective study with a parallel design of two groups. After giving informed consent, patients will not know to which group they have been assigned (reinforcement with Hemopatch or not). After the last follow-up visit on postoperative day 90, the patients can receive this information if they wish.

This study is also blind on the part of the observer; the final result will be determined by an independent observer, making this a double-blind study.

# Primary objective and secondary objectives.

**Primary endpoint:** POPF type B and C, according to the definition of the ISGPF (Bassi, Surgery 2005), that is, the secretion of pancreatic fluid externalized through a drain or removed during a reoperation, independently of the color and / or appearance on day 3 or later, whose amylase is 3 times the values of serum amylase or collections (infected or not) detected by fistulography or other imaging techniques (computed tomography, ultrasound, magnetic resonance imaging). See attached table.

| Grade | A | B | C |
| --- | --- | --- | --- |
| Clinical conditions | **Well** | **Often well** | **Ill appearing / bad** |
| Specific treatment* | **No** | **Yes / no** | **Yes** |
| US / CT (if  obtained) | **Negative** | **Negative / positive** | **Positive** |
| Persistent drainage  (after 3 weeks)** | **No** | **Usually yes** | **Yes** |
| Reoperation | **No** | **No** | **Yes** |
| Death related to  POPF | **No** | **No** | **Possibly yes** |
| Signs of infections | **No** | **Yes** | **Yes** |
| Sepsis | **No** | **No** | **Yes** |
| Readmission | **No** | **Yes / no** | **Yes / no** |
| US, Ultrasonography; CT, computed tomographic scan; POPF, postoperative pancreatic fistula.  *Partial (peripheral) or total parenteral nutrition, antibiotics, enteral nutrition, somatostatin analogue and/or minimal invasive drainage.  **With or without a drain in situ | | | |

# Secondary endpoints:

1. Duration of postoperative hospital stay.
2. Duration of stay in intensive care unit.
3. Reinterventions including interventional radiology
4. Complications in the deep/organ space up to 90 days postoperatively.
5. Delayed gastric emptying.
6. Biliary fistula.
7. Postoperative hemorrhage.
8. Death, regardless of the cause.

# Duration of the study.

The study will begin on February 1, 2018. Patients will be recruited at Miguel Servet University Hospital from January 1, 2018 to July 31, 2019. All patients randomly assigned will be followed for 30 days after surgery, or during the total stay in case it is longer than 30 days. They will also be evaluated in external consultations in the postoperative period for 3 months. A period of 4-6 months is considered to evaluate the data and the study will entail 2 years in total.

# PATIENTS.

# Criteria.

Despite improvements in morbidity after pancreatic resection, POPF after PD remains problematic. The incidence varies markedly in the literature, ranging from 0% to 64%, and 35% are clinically relevant. This great variability is attributed in part to the

indication and extension of the resection, as well as to the comorbidities of the patient. Other factors, such as age, male sex, obesity, prolonged operation time and a soft pancreas, as well as multivisceral resections increase the risk of fistula. The sequelae of pancreatic fistula after PD cover a broad spectrum. These patients often require visits to the emergency department, hospital readmissions, percutaneous drainage guided by interventional radiology, prolonged parenteral antibiotic therapy, radiological surveillance and numerous postoperative visits. The complications derived from the fistula double the cost of hospital treatment and dramatically increase the use of health resources.

# Recruitment.

All patients undergoing PD by laparotomy are potential participants. Their diagnoses include chronic pancreatitis, benign (for example, cystadenoma) or malignant tumors (adenocarcinoma, NET, metastasis, etc.). They will be invited to participate in the study and will be informed of the possible advantages and disadvantages. The randomization process will begin for any patient who agrees to participate after informed consent.

# Inclusion and exclusion criteria.

**Inclusion criteria:** patients submitted to PD by open approach for benign/malignant periampullary tumours or benign; ASA < 4; men and women ≥ 18 and ≤ 80 years of age.

**Exclusion criteria:** patients under 18 and over 80 years old; ASA score ≥ 4; subjected to multivisceral resections different from PD; patients with acute necrotizing pancreatitis; immunosuppressed patients; patients who have not signed the informed consent; patient with contraindication of PD performance; vascular reconstruction; neoadjuvant therapy.

# Intervention techniques and materials.

The indications for performing a PD are multiple. The type of reconstruction to be performed will depend basically on the characteristics of the pancreatic remnant. However, in our center, the usual reconstruction technique is the duct-to-mucosa type pancreatojejunostomy.

Depending on the randomization, patients will be operated as follows:

1. Basic treatment: standard duct-to-mucosa pancreatojejunostomy.
2. Basic treatment plus Hemopatch: standard pancreatojejunostomy + reinforcement with 2 Hemopatch of 90 x 45 mm, one on each side (lower and upper) as a "scarf".

In both groups, passive external drainage is mandatory for a period of 7 days with drainage placed next to the anastomosis but not in contact with it. The drain can be a Penrose drain, a Jackson-Pratt drain or Blake without active suction. Suction drains are not allowed.

Adjuvant procedures

Wrapping, sealing with fibrin or serous patch with another adjacent organ are not acceptable. No resection of adjacent hollow organ is allowed (excepting those included in the standard DPC).

Materials

Hemopatch is a hemostatic-sealant made of collagen that resorbs in 6-8 weeks. The active side is covered with a polyethylene glycol film for better adhesion.

Hemopatch is a medical product with CE certificate (Annex IV - excluding section 4 of Council Directive 93/42 / EEC on medical devices) used for hemostasis. It is not designed for use with heavy or pulsatile bleeding or with acute infections. It also has indication of sealant from March 2016. Hemopatch should not be used intravascularly. It also can not be used for patients who are allergic to any of the materials listed by the manufacturer. The only known side effect is the rare case when Hemopatch can not achieve the desired hemostasis. With documentation of the patient's clinical course, regular blood tests and imaging tests when necessary, any undesirable effects derived from the use of Hemopatch must be diagnosed in a timely manner and appropriate measures will be taken, the coordinator will be immediately informed, the person in charge of the control of the test and, likewise, an immediate communication of the adverse effect to the manufacturer will be sent.

# Postoperative period.

In the postoperative period, the parameters of inflammation, body temperature, visual analog pain scale (VAS), drainage debit and its amylase / lipase content will be documented. To facilitate the healing process, drainage should be left in place for at least 7 days. In the absence of evidence of fistula, the drainage may be withdrawn. If the 7th postoperative day there is evidence of pancreatic fistula according to the ISGPF criteria, the drainage will be maintained until the resolution of the same.

All patients are advised to follow the instructions given at discharge. If there is any reason for the alarm (pain, fever, tachycardia) the patient should go to the hospital where he was treated at any time. A final visit will be scheduled on postoperative day 30 to evaluate any collection of intra-abdominal fluids through the clinical examination and the performance of an ultrasound or CT scan (as is done routinely).

# Complications / failure of the intervention.

POPF are defined according to the definition published in 2005 by the International Study Group on Pancreatic Fistula (ISGPF), which is based on the concentration of amylase in the drainage fluid. A POPF is defined when the concentration of amylase in the drainage fluid is three or more times greater than in serum from the third postoperative day.

Pancreatic fistulas are classified in 3 grades, from biochemical leakage to C:

- - - **BIochemical leakage:** clinically asymptomatic patient, persistent fistula, absence of intra-abdominal fluid collection in imaging test. No therapeutic consequence; the hospitalization is not prolonged.
    - **Grade B:** symptomatic patient, requires diagnostic evaluation and therapeutic management. Clinically stable patient, may complain of abdominal pain, fever, nausea and intolerance to oral intake. Normally there is a collection of at least 3 x 3 cm in image tests. Therapeutic consequences: you must start antibiotic treatment and absolute diet. Invasive intervention (percutaneous drainage) may be necessary; hospitalization usually lasts.
    - **Grade C:** clinically unstable patient (sepsis). Therapeutic consequences: it requires intensive care, percutaneous drainage or reoperation if the drainage has been removed or is not properly positioned. Hemorrhage is frequent; high expected mortality.

The objective of this study is to evaluate the rate of type B and C fistulas after pancreatic resection with and without Hemopatch.

# Termination conditions / Adverse events / Suspected unexpected serious adverse reaction.

Criteria for withdrawing a patient from the study:

- - - Patient's desire to be removed from the study.
    - Intervention not performed (due to unresectability).
    - The investigator suspects a serious threat to the patient's well-being. Adverse events (any sign or symptom that affects the patient's well-being) and serious adverse events (life-threatening, prolongs hospital stay, results in persistent disability or leads to death during the observation period), as cardiopulmonary problems, in the gastrointestinal tract, anaphylactic shock or embolism will be recorded through daily postoperative visits until discharge and during the final visit on the 30th postoperative day. All adverse effects of clinical relevance and all serious adverse effects will be recorded in the case report form along with the degree of the complication (according to the Clavien-Dindo classification). The principal investigator must be informed within 24 hours about the serious adverse effects that occur during the hospital stay. The presence of a causal relationship between adverse affect or serious adverse effect and the intervention of the trial will be judged by the investigator. Serious adverse effects will be reported immediately (ie, within 5 days after recognition) by all investigators to the coordinating investigator. The coordinating researcher must inform the responsible ethics committee and the research sponsor. A list of all serious adverse effects must be compiled.

# DATA.

- 1. **Calculation of the sample size.**

An observational study was carried out for patients undergoing PD in our institution and indicated a baseline 31% CR-POPF rate. According to power calculations, 62 patients (31 per group) were required to show decrease of CR-POPF rate from 31% to 5% with 80% power and two tailed 5% significance level, with assumed 10% loss-to-follow-up.

# Randomization and groups.

Patients will be randomized on a 1:1 basis during surgery by the minimization method (preferred treatment probability 0.9), after resection but before anastomosis, to either PEG-coated patch application (Patch group) or no patch (Control group). Randomization will be done by blocks of 10 patients, using a secure, internet-based, randomization service (Randomizer®), provided by the Centre of Medical Statistics, Medical University of Graz, Austria^33^, to all participating sites. Randomization will be stratified by pancreatic gland texture (soft or normal), pancreatic duct diameter (up to 3 mm or larger than 3 mm) and intraoperative bleeding (up to 700 cc or more than 700 cc).

Patients will be assigned to one of the following study groups: Patch group: Patients undergoing PD with PJ reinforced with two PEG-coated collagen-based haemostatic patches; and Control group: Patients undergoing PD with PJ without any sealant reinforcement. Although operating-room staff and doctors responsible of the follow-up were no blind to the intervention, data analysis will be performed in a masked fashion.

# Efficacy assessments (that is, data that must be collected).

- - - Daily drainage debit.
    - Concentration of amylase and lipase from the drainage fluid (days 3, 5 and 7 postoperatively) and every 2 days in case of permanent fistula.
    - Number of postoperative days with drainage (minimum 7 days). If the amylase / lipase content of the drainage on the 7th postoperative day is within normal limits, it may be withdrawn.
    - Body temperature, VAS pain score, leukocyte count and CRP.
    - Duration of the stay in the ICU.
    - Duration of the total hospital stay.
    - Reinterventions, including percutaneous drainage of collections.
    - Re-admission.
    - Degree of complications according to the Clavien-Dindo classification, including both complications derived from surgery (delay of gastric emptying, bleeding, biliary fistula, wound infection, intra-abdominal

abscess, etc.) and general (atelectasis, pneumonia, urine infection, thromboembolism, etc.)

# Patient data sheet.

It contains patient information including personal history (ie, smoking, diabetes, BMI), preoperative and postoperative data, intraoperative incidents, special treatment details, complications and adverse events such as new onset diabetes, pneumonia or heart problems.

All the data are collected in a central database.

All study personnel must sign a confidentiality agreement that covers all aspects of the study and its data. The study directors will take particular responsibility for the security of the data, the documents of origin and the information about the patients.

The coordinator of the main study is also responsible for continuing education in Good Clinical Practices (GCP) in accordance with the Declaration of Helsinki.

# Statistical methods and data analysis.

Statistical analyses will be performed using R software (version 4.0.3). Descriptive statistics number (percentage) or median [interquartile range] will be used for categorical and continuous variables, respectively. Data were tested for normal distribution using the Shapiro-Wilk test. The Chi^2^ with Yates correction or the independent sample Mann-Whitney U-test were used for comparing categorical and continuous variables, respectively.

A multivariable logistic regression model will be used to estimate the odd ratios and the 95% confidence intervals (CIs) associated with the use of patches and CR-POPF. Postoperative pancreatic fistula survival rates will be plotted for treatment groups using Kaplan–Meier analysis and were compared using a log-rank test. A p-value of less than 0.05 was considered significant, and all tests were two-tailed.

# PATIENT SAFETY.

A monitoring team will receive all patient data electronically including expected and unexpected events and side effects for all patients in the study. All patient data are analyzed to detect any unusual pattern. There will be permanent contact with the main coordinator of the study and will meet face to face three times a year to evaluate the course of the study based on the data collected.

# IMPACT ON CLINICAL PRACTICE.

Hemopatch is expected to reduce the risk of postoperative type B and C pancreatic fistula from 30% to 15%, so that the number of patients currently requiring readmission with possible surgeries and interventional radiological procedures is halved. This implies not only clinical but also economic advantages, since a pancreatic fistula can double the cost of hospital treatment.

# ETHICS AND LEGAL REQUIREMENTS.

This study is carried out in full compliance with the principles of the "Declaration of Helsinki" (amended at the 56th General Assembly of the AMM, Tokyo, Japan, 2008). Following good clinical practices should guarantee the scientific conduct of the clinical trial and the credibility of the results. This study is approved by the Clinical Research Ethics Committee of Aragon (CEICA).

# REGISTRATION.

Once approved, this study will be registered in the EU Clinical Trials Register or ClinicalTrials.gov.

# PUBLICATION.

Once the study is completed and the statistical study has been carried out, a publication will be made and sent to a high-impact surgical journal in English.

# REFERENCES.

1. Yoshioka R, et al. Impact of hospital volume on hospital mortality, length of stay and total costs after pancreaticoduodenectomy. Br J Surg 2014; 101: 523–9.
2. Kimura W, et al. A pancreaticoduodenectomy risk model derived from 8575 cases from a national single-race population (Japanese) using a web-based data entry system: the 30-day and in-hospital mortality rates for pancreaticoduodenectomy. Ann Surg 2014; 259: 773–80.
3. Winter JM, et al. 1423 pancreaticoduodenectomies for pancreatic cancer: a single-institution experience. J Gastrointest Surg 2006; 10: 1199–210.
4. Schmidt CM, et al. Pancreaticoduodenectomy: a 20-year experience in 516 patients. Arch Surg 2004; 139: 718–25.
5. Glazer ES, et al. Recognition of complications after pancreaticoduodenectomy for cancer determines inpatient mortality. JOP 2013; 14: 626–31.
6. De Oliveira ML, et al. Assessment of complications after pancreatic surgery: a novel grading system applied to 633 patients undergoing pancreaticoduodenectomy. Ann Surg 2006; 244: 931–7.
7. Menahem B, et al. Pancreaticogastrostomy is superior to pancreaticojejunostomy for prevention of pancreatic fistula after pancreaticoduodenectomy: an updated meta-analysis of randomized controlled trials. Ann Surg 2015; 261: 882–7.
8. Hua J, et al. Duct-to-mucosa versus invagination pancreaticojejunostomy following pancreaticoduodenectomy: a systematic review and meta- analysis. J Gastrointest Surg 2015; 19: 1900–9.
9. Gurusamy KS, et al. Somatostatin analogues for pancreatic surgery. Cochrane Database Syst Rev 2013; 4:CD008370.
10. Zhou Y, et al. The impact of internal or external transanastomotic pancreatic duct stents following pancreaticojejunostomy. Which one is better? A meta- analysis. J Gastrointest Surg 2012; 16: 2322–35.
11. Gómez T, et al. Reconstruction after pancreatoduodenectomy: Pancreatojejunostomy vs pancreatogastrostomy. World J Gastrointest Oncol 2014; 6(9): 369-76.
12. Keck T, et al. Pancreatogastrostomy versus Pancreatojejunostomy for RECOnstruction After PANCreatoduodenectomy (RECOPANC, DRKS 00000767) Perioperative and Long-term Results of a Multicenter Randomized Controlled Trial. Ann Surg 2016; 263(3): 440-9.
13. Peng SY, et al. Conventional versus binding pancreaticojejunostomy after pancreaticoduodenectomy: a prospective randomized trial. Ann Surg 2007; 245: 692–8.
14. Bassi C, et al. Pancreatic fistula rate after pancreatic resection. The importance of definitions. Dig Surg 2004; 21: 54–9.
15. El Nakeeb A, et al. Pancreatic anastomotic leakage after pancreaticoduodenectomy. Risk factors, clinical predictors, and management (single center experience). World J Surg 2013; 37: 1405–18.
16. Jang JY, et al. Randomized multicentre trial comparing external and internal pancreatic stenting during pancreaticoduodenectomy. Br J Surg 2016; 103(6): 668-75.
17. Lillemoe KD, et al. Does fibrin glue sealant decrease the rate of pancreatic fistula after pancreaticoduodenectomy? Results of a prospective randomized trial. J Gastrointest Surg 2004; 8:766–72.
18. Ochiai T, et al. Application of polyethylene glycolic acid felt with fibrin sealant to prevent postoperative pancreatic fistula in pancreatic surgery. J Gastrointest Surg 2010; 14: 884–90.
19. Pozzo G, et al. A new method of jejunal reconstruction after pancreaticoduodenectomy. Hepato-gastroenterology 2010; 57 (102-103): 1305- 8.
20. Mita K, et al. Pancreaticojejunostomy using a fibrin adhesive sealant (TachoComb) for the prevention of pancreatic fistula after pancreaticoduodenectomy. Hepato-gastroenterology 2011; 58 (105): 187-91.
21. Satoi S, et al. Reinforcement of Pancreticojejunostomy Using Polyglycolic Acid Mesh and Fibrin Glue Sealant. Pancreas 2011; 40 (1): 16-20.
22. Martin I, et al. Does fibrin glue sealant decrease the rate of anastomotic leak after a pancreaticoduodenectomy? Results of a prospective randomized trial. HPB 2013; 15 (8): 561-6.
23. HEMOPATCH - Instructions for use. Baxter.
24. Bassi C, et al. Postoperative pancreatic fistula: An international study group (ISGPF) definition. Surgery 2005; 138(1): 8-13.

**Supplementary Tables**

**Table S1. POPF rates according to the technique used.**

|  | **Overall**  **(n=64)** | **Interrupted suture**  **(n=8)** | **Continuous suture**  **(n=56)** | **p** | **N** |
| --- | --- | --- | --- | --- | --- |
| **Overall POPF, n (%)** | 24 (38) | 2 (25) | 22 (39) | 0.699 | 64 |
| **Type of POPF, n (%)**  BL  B  C | 15 (63)  4 (17)  5 (21) | 2 (100)  0 (0)  0 (0) | 13 (59)  4 (18)  5 (23) | 1.000 | 24 |
| **CR-POPF, n (%)** | 9 (14) | 0 (0) | 9 (16) | 0.587 | 64 |

POPF= Postoperative pancreatic fistula; BL= Biochemical leakage; CR-POPF= Clinically relevant postoperative pancreatic fistula
